# Microsurgical complication associated with vaccine-induced immune thrombotic thrombocytopenia (VITT): A case report

**DOI:** 10.1097/MD.0000000000033013

**Published:** 2023-02-17

**Authors:** Chen-Ting Hung, Honda Hsu

**Affiliations:** a Hualien Tzu Chi Hospital, Buddhist Tzu Chi Medical Foundation, Hualien, Taiwan; b Dalin Tzu Chi Hospital, Buddhist Tzu Chi Medical Foundation, Dalin, Taiwan; c School of Medicine, Tzu Chi University, Hualien, Taiwan.

**Keywords:** case report, free flap, VITT

## Abstract

The use of ChAdOx1 nCoV-19 (Astra Zeneca) vaccine has proven beneficial, but in a limited number of the general population, it was found to be associated with vaccine-induced immune thrombotic thrombocytopenia (VITT). However, there have been no reports of this complication occurring in a microsurgical free tissue transfer.

**Patient concerns:**

A 49-year-old man developed an acute myocardial infarction 3 weeks after receiving his first dose of ChAdOx1 nCoV-19 in June 2021. Three months later, he presented with right third toe wet gangrene with extension into the plantar foot nine days after receiving his second dose of ChAdOx1 nCoV-19 vaccine.

**Diagnosis:**

Based on recent exposure to vaccination, the timing of inoculation before the development of his symptoms, and serology tests (platelet, D-dimer, and anti-PF4 antibodies), the patient was diagnosed with VITT.

**Interventions:**

Fasciectomy and sequestrectomy were performed for wound bed preparation. Limb salvage was done using free vastus lateralis muscle flap and skin graft for reconstruction.

**Outcome:**

The flap was complicated by persistent microthrombi leading to superficial necrosis without vascular pedicle compromise. Repeated debridement of the superficial necrosis was done. Three months after the development of VITT, no further new superficial necrosis was seen. A well-contoured flap was seen 5 months after the initial surgery.

**Lessons:**

We believe this is the first case describing microthrombi in the free flap due to VITT after microsurgical reconstruction. Patients and surgeons should be advised of this possible risk when contemplating microsurgery once VITT has developed after ChAdOx1 nCoV-19 administration.

## 1. Introduction

The coronavirus disease 2019 (COVID-19) pandemic, due to severe acute respiratory syndrome coronavirus 2 (SARS-CoV-2) infection, has caused significant morbidity and mortality globally. As of early May 2022, there have been >500 million confirmed cases of COVID-19, including over 6 million deaths, reported to the World Health Organization. In order to prevent SARS-CoV-2 infection, several types of vaccine were made available to be used after approval by the Emergency Use Authorization Act in various countries since the end of 2020. However, different types of adverse effects and complications caused by the vaccines were soon reported.^[1]^ Vaccine-induced immune thrombotic thrombocytopenia (VITT) was one of the more lethal complications reported. This syndrome was specifically related to the SARS-CoV2 adenoviral vector vaccine. It causes symptoms, such as headache, visual changes, persistent chest pain, or lower limb swelling according to different thrombotic events that have happened. Pavord et al^[2]^ in 2021, found that in 220 cases with VITT in the UK, the overall mortality reached 22%. The most common sites of thrombosis are the cavernous sinus, the pulmonary artery, and the deep veins in the lower limbs. Currently, there have been no reports of this complication occurring in a microsurgical free tissue transfer.

## 2. Case presentation

**Figure 1.**
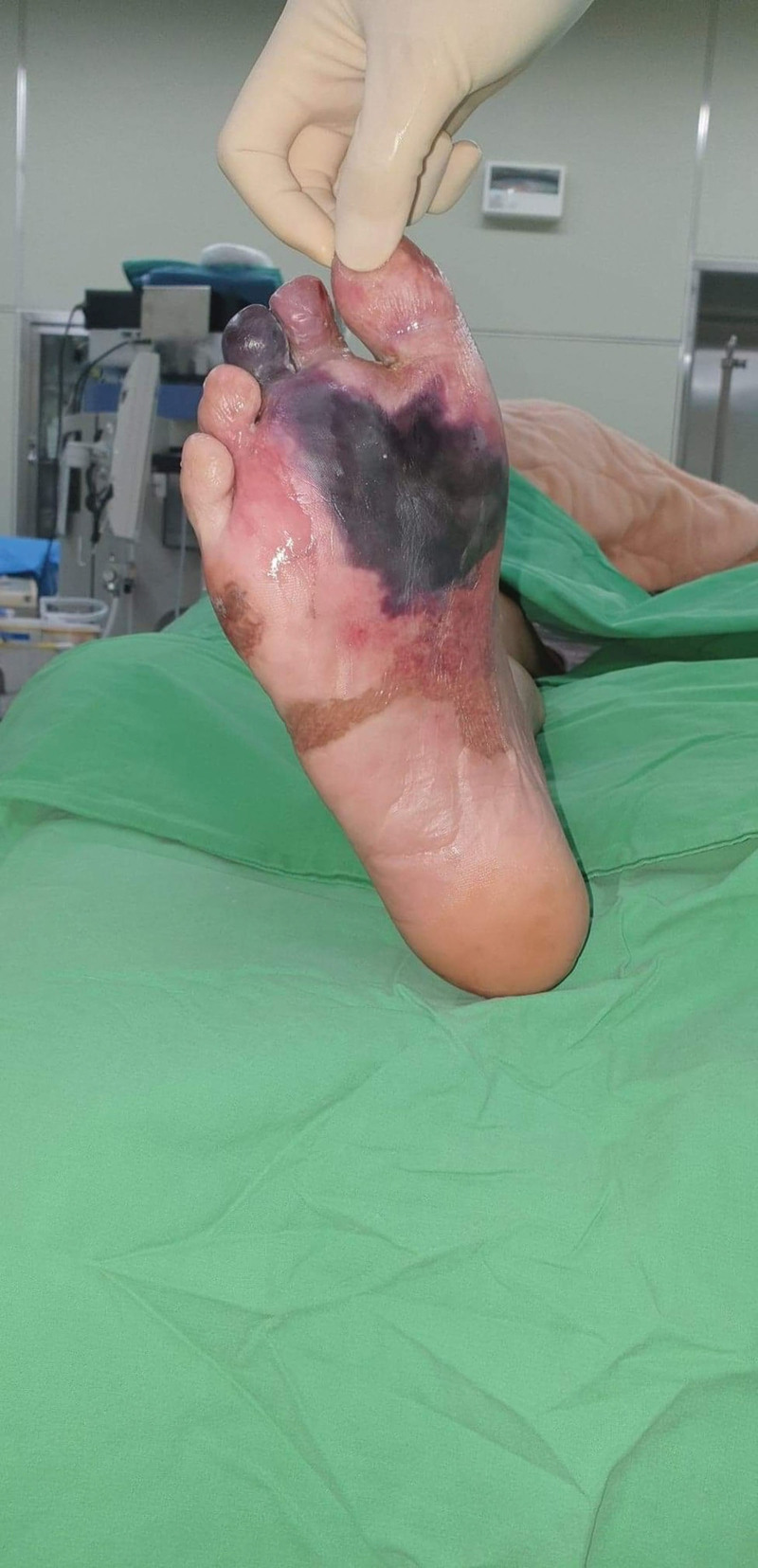
Right third toe wet gangrene with extension into the plantar foot after receiving the second dose of ChAdOx1 nCoV-19 vaccine.

**Figure 2.**
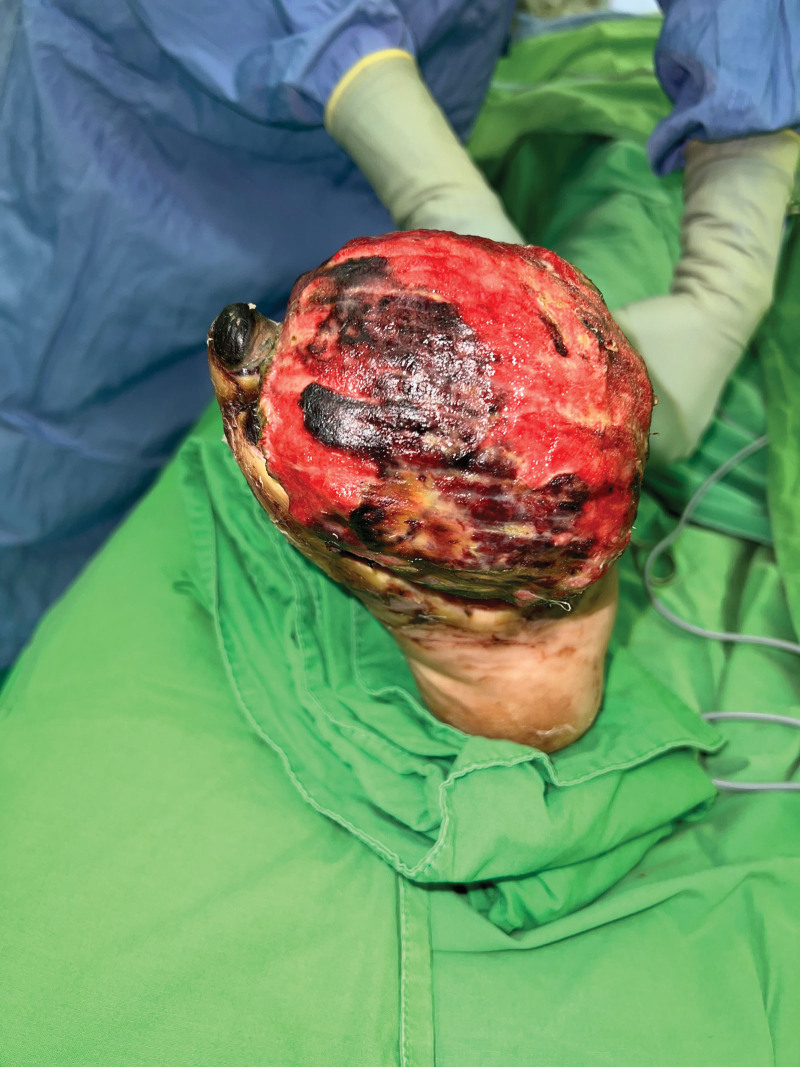
The flap was complicated by persistent partial necrosis of the superficial area without the vascular pedicle compromised.

**Figure 3.**
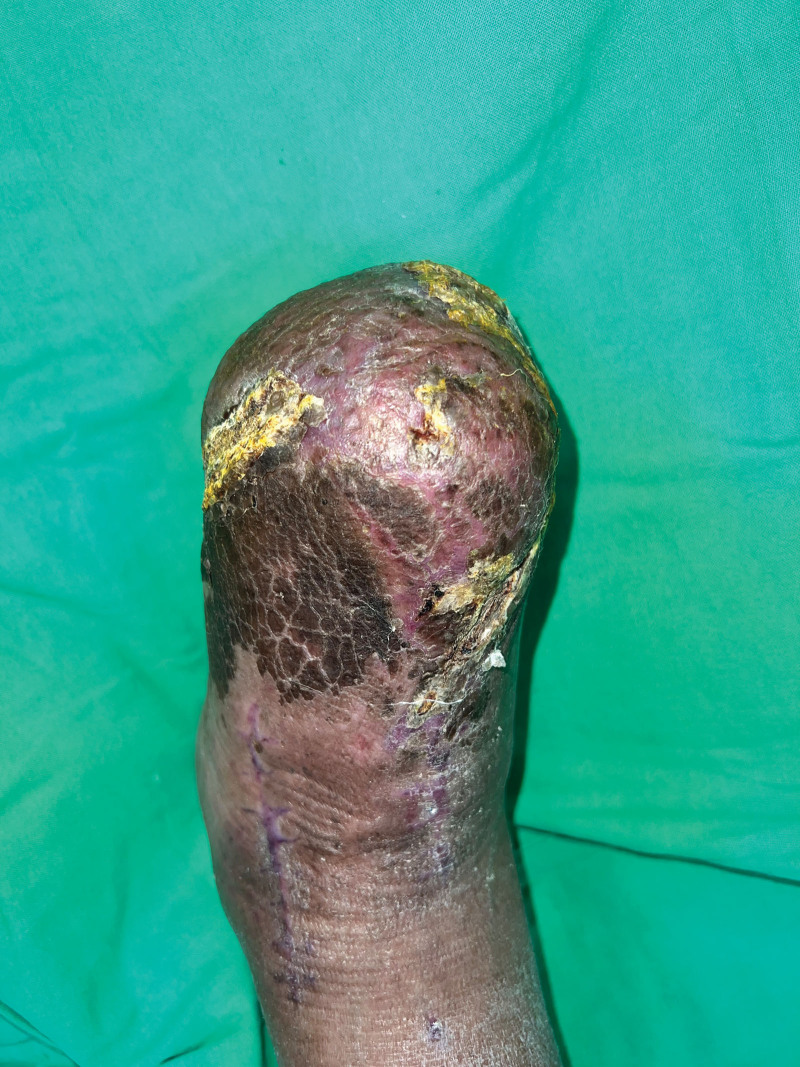
No further superficial necrosis of the flap was seen after repeated tangential debridement.

**Figure 4.**
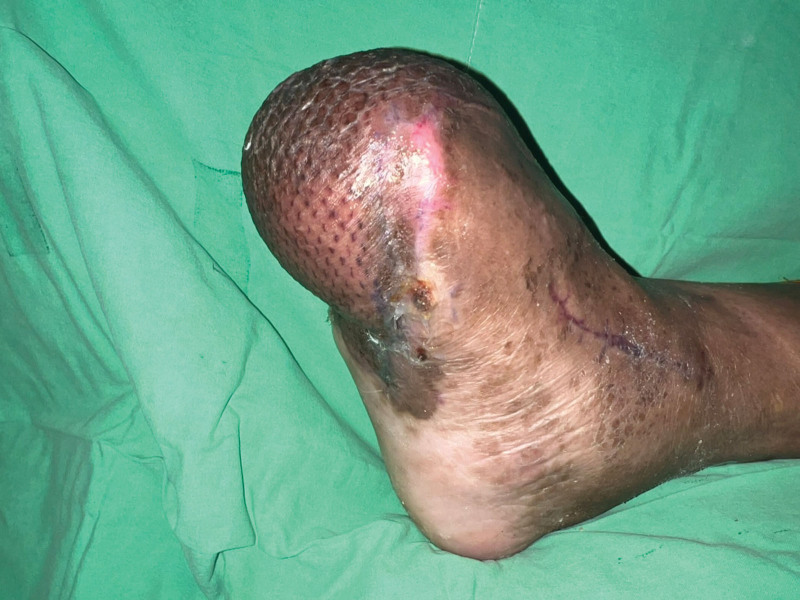
A well-contoured flap was seen 5 months after the initial surgery.

A 49-year-old man with a history of hypertension, diabetes mellitus, and end-stage renal disease on hemodialysis received his first dose of ChAdOx1 nCoV-19 (Oxford–AstraZeneca, AZ) on June 17, 2021. Three weeks later, he complained of chest discomfort and the cardiac echo showed dilated left ventricle chamber, anteroseptal wall hypokinesis with apical dyskinesis, and impaired left ventricle systolic function. Cardiac catheterization was arranged and 3-vessel disease was found. Coronary artery bypass surgery was performed on July 14 and the patient was discharged in early August. He recovered uneventfully. He received his second dose of the ChAdOx1 nCoV-19 (Oxford–AstraZeneca, AZ) vaccine on October 16, 2021. Nine days later, he presented to the Emergency Room for right third toe wet gangrene with extension into the plantar foot (Fig. 1). According to the patient, right foot erythema and mild fever were noted 2 days after the second AZ vaccination. Initially, he did not pay much attention but the lesion progressively deteriorated. On presentation at the Emergency Department, his full blood count showed an elevated white blood cell count of 10,270 per µL and the C-reactive protein was also raised at 13.06 µg per dL. Emergent fasciotomy was performed. Poor blood flow was seen in the wound bed. Due to the timing of inoculation of the vaccine and his symptoms, VITT was considered. Further, the work-up showed his platelet count to be 140 × 10^3^/µL, D-dimer was 4916 ng/mL and anti–platelet factor 4 (PF4) antibodies were 83.77 ng/mL (reference value for positive, >50). According to the International Society on Thrombosis and Hemostasis interim guidance, a definite diagnosis of VITT was made. Conservative treatment without oral anticoagulant, intravenous immunoglobulin, or therapeutic plasma exchange was done, as the patient did not show any signs of headache, chest tightness or dyspnea and he did not have a trend of decreasing platelet count and fibrinogen levels. Due to progressive necrosis of the forefoot, the forefoot amputation was done with the 5th toe spared, as he was adamant that he did not want it amputated as the toe looked fine to him. Due to a resultant large soft tissue defect with bone exposure, a free vastus lateralis muscle flap with split-thickness skin graft was performed for limb salvage. The flap survived well and he was discharged 1 month later. This patient was regularly followed up at our outpatient clinics for wound care. Unfortunately, right 5th toe gangrene and partial dehiscence at the lateral plantar side of the right foot were noted, so he was admitted again for surgical debridement and amputation of the 5th toe. This time he agreed to the amputation of the 5th toe. The flap had survived well but was complicated by persistent partial necrosis of the superficial portion of the flap without vascular pedicle compromise. A possible etiology causing this was vaccine-induced microthrombi (Fig. 2). After repeated debridement and when no further necrosis was seen (Fig. 3), the flap was skin grafted for final coverage. A well-contoured flap was seen 5 months after the initial surgery (Fig. 4). Written informed patient consent was given by the patient, as well as institutional review board approval was obtained for the publication of this case report.

## 3. Discussion

The use of microsurgical free tissue transfer for extensive soft tissue defects of the lower limb is a commonly performed procedure for lower limb salvage.^[3]^ The main complication after free tissue transfer is often due to thrombosis at the vascular anastomotic site. Microthrombi within the flap are not commonly encountered.

In this case, peripheral artery occlusive disease could have been the main primary etiology of the right foot gangrene. But VITT was a strong contributing factor due to the events that occurred after receiving both doses of ChAdOx1 nCoV-19 (Oxford–AstraZeneca, AZ) vaccine. After debridement, we chose a free muscle flap for reconstruction as we felt that providing a highly vascularized soft tissue with a low resistant bed would provide a better run-off for the lower limb vessels as well as act as a nutrient flap for the wound. In the second admission, the flap had survived well with no signs of an infective process. But partial necrosis of the superficial layer of the muscle flap was noted even though both the arterial and venous pedicle was patent. This was different from what we usually encounter for partial flap or total flap necrosis. Often the cause of flap necrosis is thrombosis of the pedicle, either the artery or the vein; or thrombosis of a branch of the vessel from the main pedicle leading to a portion of the flap that becomes necrotic. Infection is also a cause of partial flap necrosis. But in all of these situations, we do not encounter superficial necrosis of the flap in a random pattern.

Although the mechanism of VITT is still not certain, it can cause insidious thrombotic events in the microcirculation apart from thrombosis in the sites previously mentioned. Fanni et al^[4]^ described autopsy cases after VITT, showing microthrombi in the capillaries and veins of the lungs, glomerular tufts, liver, vasa vasorum of the aorta, and brain. Althaus et al^[5]^ reported 3 cases where autopsy also revealed arterial and venous microthrombi in various organs. This could be the same mechanism leading to microthrombi in our free flap.

Astra Zeneca vaccine, or ChAdOx1 nCoV-19 vaccine, is categorized as a viral vector vaccine. The chosen viral vector is a chimpanzee adenovirus which is a replication-deficient virus. It was engineered to express the genetic sequence of the spike protein from SARS-CoV-2 in host cells which then triggers sequential active immunity.^[6]^ The effectiveness of the AZ vaccine was satisfactory according to the pooled interim analysis published by Voysey et al, and the overall vaccine efficacy was 70.4%.^[7]^ However; there were several articles that reported a rare but lethal complication, VITT, related to the AZ vaccine. The incidence rate was estimated to be 0.87 cases per million doses.^[8]^

The hypothetical pathogenesis of VITT after ChAdOx1 nCoV-19 vaccination is believed to be related to FcγRIIa (CD32a) receptors.^[9]^ Bye et al have proposed the hypothesis by in vitro fundamental study. They found that aberrant glycosylation of the heavy chain of the anti-SARS-CoV-2 IgG increased galactosylation instead of fucosylation. This enhances the binding affinity to the FcγRIIa on the platelet.^[10]^ This in turn triggers increased von Willebrand factor production by signaling transmission. This is believed to be the cause of the pro-thrombotic effect. The incidence rate of VITT was estimated to be 0.87 cases per million doses.^[8]^

The duration of the thrombogenic effect of VITT is also unclear. In our patient, the phenomenon lasted for five months at which time, no further microthrombi was seen and final reconstruction with skin graft could be undertaken. Schönborn et al^[11]^ in a 65-case VITT cohort study, found that all the patients who showed positive anti-PF4 antibodies in the acute phase also showed that titers of antibodies were still detectable by enzyme immunoassay 6 months (84.2%) and 9 months (78.6%) after vaccination. At the same time, 3 out of the 26 patients still showed recurrent episodes of VITT in whom anti-PF4 antibodies persisted for at least 3 months. The relationship between persistently elevated anti-PF4 antibodies and the pathogenesis of VITT is still unclear. Since VITT is still a novel syndrome after COVID-19 vaccination, further research into the duration of the thrombotic event and the relationship with the titer of antibodies are still required.

## 4. Conclusion

In the COVID-19 pandemic era, several types of vaccines were available to be used rapidly and commonly in order to prevent the SARS-CoV-2 infection. VITT was one of the more lethal complications associated with the vaccines. We believe this is the first case describing microthrombi in the free flap due to VITT after microsurgical reconstruction. Patients and surgeons should be advised of this possible risk when contemplating microsurgery once VITT has developed after administration.

## Author contributions

**Conceptualization:** Chen-Ting Hung, Honda Hsu.

Supervision: Honda Hsu.

Validation: Chen-Ting Hung.

Writing – original draft: Chen-Ting Hung.

Writing – review & editing: Honda Hsu.
